# Importance of Central Retinal Sensitivity for Prediction of Visual Acuity after Intravitreal Bevacizumb in Eyes with Macular Edema Associated with Branch Retinal Vein Occlusion

**DOI:** 10.1371/journal.pone.0149246

**Published:** 2016-02-17

**Authors:** Masahiko Sugimoto, Atsushi Ichio, Mineo Kondo

**Affiliations:** Department of Ophthalmology, Mie University Graduate School of Medicine, Tsu, Japan; Tohoku University, JAPAN

## Abstract

**Objective:**

To determine whether the baseline retinal sensitivity can predict the best-corrected visual acuity (BCVA) at 1 month after intravitreal bevacizumab (IVB) in eyes with macular edema (ME) associated with a branch retinal vein occlusion (BRVO).

**Subjects and Methods:**

We evaluated 16 eyes of 16 patients who had ME associated with a BRVO. The mean ± standard deviation age was 69.1 ± 8.9 years, and all had a single IVB injection. The BCVA, central macular thickness (CMT), integrity of the ellipsoid zone (EZ) of the photoreceptors, and retinal sensitivity were determined before (baseline) and at 1 day, 1 week, and 1 month following the IVB. The average threshold retinal sensitivity (AT) within the central 10° was determined by Macular Integrity Assessment. The correlations between the BCVA at 1 month and the CMT, integrity of the EZ, and AT at each visit were determined.

**Results:**

One month after IVB, the BCVA improved significantly from 0.56 ± 0.27 logMAR units to 0.32 ± 0.28 logMAR units, and the CMT from 611.4 ± 209.3 μm to 258.7 ± 64.0 μm (*P* <0.05). The AT improved significantly from 17.9 ± 5.3 dB to 21.2 ± 5.0 dB (*P* <0.05). At 1 day after the treatment, both the integrity of the EZ (r = 0.59) and the retinal sensitivity (r = 0.76) were moderately correlated with the BCVA at 1 month.

**Conclusion:**

These results indicate that both the integrity of the EZ and the AT at 1 day after the IVB can predict the BCVA after treatment for ME associated with BRVO. There is a possibility that these parameters will predict the effectiveness of IVB for each case.

## Introduction

Macular edema (ME) is the main cause of visual impairments in eyes with branch retinal vein occlusion (BRVO). The increased hydrostatic pressure caused by the occlusion leads to an acute extravasation of fluid into the extracellular space of the retina which is detected as ME [1]. There is also an up-regulation of vascular endothelial growth factor (VEGF) in response to the hypoxia which leads to a breakdown of the blood–retinal barrier [2, 3].

Various therapies have been developed to treat the ME associated with a BRVO. Anti-VEGF drugs and steroids are used as standard treatments because they alleviate the disruption of the blood–retinal barrier which results in an improvement of the degree of ME [4–8]. Treatment by a single therapeutic procedure does not always improve the vision and reduce the degree of ME, and this failure is important because prolonged ME can cause severe damage of the outer layers of the retina leading to irreversible damage. Thus, it is important to determine the effectiveness of an ongoing therapy and decide when it is appropriate to switch to other therapeutic procedures.

Optical coherence tomography (OCT) enables clinicians to evaluate the morphological characteristics of the retina. Although morphological analyses of different retinal diseases by OCT has greatly improved [9–11], the improvements do not always indicate functional improvements [12]. Thus, while the imaging techniques are helpful for clinicians, the functional parameters still need to be examined.

It has still not been definitively determined how to predict the final visual acuity after therapy although the results of many studies have been reported on different preoperative morphological and physiological parameters that might be used. The retinal sensitivity determined by microperimetry is one of the physiological parameters that has been considered. The retinal sensitivities allow clinicians to document changes in the pericentral sensitivity for the diagnosis and follow-up of eyes with macular diseases [13–18].

The Macular Integrity Assessment (MAIA^™^, Center Vue, Padova, Italy) instrument is a scanning laser ophthalmoscope that incorporates a high speed eye tracker and can measure the sensitivity in the macular area [19]. Because of the eye tracker software, the sensitivity of the same area of the retina can be determined repeatedly. This will allow clinicians to follow the effect of different types of therapy on the retinal sensitivity in the same area. A search of MEDLINE did not extract any publications that used MAIA to follow the changes in retinal sensitivity in eyes with ME due to a BRVO before and after IVB treatment.

Thus, the aim of this study was to determine whether the retinal sensitivity determined by MAIA^™^ can predict the BCVA at 1 month after a single intravitreal injection of bevacizumab (IVB) injection in eyes with ME associated with a BRVO.

## Patients and Methods

### Patients

This was a prospective study of 16 eyes of 16 consecutive patients that had ME associated with a BRVO and had been treated with IVB between May 2012 and July 2013. There were 10 men and 6 women whose mean age was 68.4 ± 9.3 years, and all were examined at the Department of Ophthalmology, Mie University Hospital. The off-label use of bevacizumab was explained to all patients, and all patients provided a signed written informed consent. Because no other anti-VEGF drugs including ranibizumab were approved before 2014 in Japan, bevacizumab was the only drug we could use at that time. The consent form also included a statement that the medical findings could be used for future research. The procedures used in this study were approved by the Institutional Ethics Review Board of the Mie University Hospital (#702) and adhered to the tenets of the Declaration of Helsinki. All methods described here were carried out in accordance with the approved guidelines. The off-label use of bevacizumab was explained to all patients, and all patients provided a signed written informed consent.

Each patient received a comprehensive ophthalmologic examination including measurements of the best-corrected visual acuity (BCVA) and intraocular pressures (IOPs), examination of the anterior segment by slit-lamp biomicroscopy, fundus examination by indirect ophthalmoscopy, and macular evaluations by optical coherence tomography (OCT) and microperimetry with the MAIA. The measurements were made before, and at 1 day, 1 week, and 1 month after the IVB.

The inclusion criteria were eyes with ME due to a BRVO, a foveal thickness greater than 300 μm, and no other retinal diseases. The exclusion criteria were prior ocular surgery, macular laser photocoagulation, and intravitreal treatment with a steroid. In addition, eyes with ocular inflammation, drusen, diabetic retinopathy, severe retinal hemorrhage which involved the intra- or sub-foveal spaces, epiretinal membrane, and glaucoma were excluded.

### Intravitreal bevacizumab injection (IVB)

IVB was injected under local subconjunctival anesthesia. Each patient received 1.25 mg of bevacizumab intravitreally, and the needle was inserted 4 mm posterior to the corneal limbus under sterile conditions. All patients received topical antibiotics for 1 week after the injection.

### Measurement of best-corrected visual acuity (BCVA)

The BCVA was measured with a Landolt chart at every visit. The decimal BCVA was converted to the logarithm of the minimum angle of resolution (logMAR) units for the statistical analyses.

### Optical Coherence Tomography (OCT)

The degree of macular edema was determined by a Heidelberg Spectralis OCT instrument (Heidelberg Engineering Inc, Heidelberg, Germany). For qualitative and quantitative analyses of the OCT images, the fast macula protocol was used to obtain the images with an automatic real time mean value of 9 which acquired 25 horizontal lines consisting of 1024 A-scans per line. The central macular thickness (CMT) was defined as the thickness between the internal limiting membrane and the retinal pigment epithelium at the fovea, and the value was automatically calculated by a bundled software.

The integrity of the ellipsoid zone (EZ) in the horizontal OCT images at the fovea was classified into three grades: Grade 1, absent; Grade 2, abnormal or difficult to identify; and Grade 3, present or continuous ellipsoid zone. All of the grading was done by one observer (MS) [10].

### Microperimetry

Fundus-monitored microperimetry was done with the MAIA^™^ scanning laser ophthalmoscope. This instrument consists of an infrared fundus camera with a software that can track eye movements automatically with respect to a reference frame obtained at the beginning of the measurements. Thus, the same area of the retina was measured at each examination.

**Fig 1:**
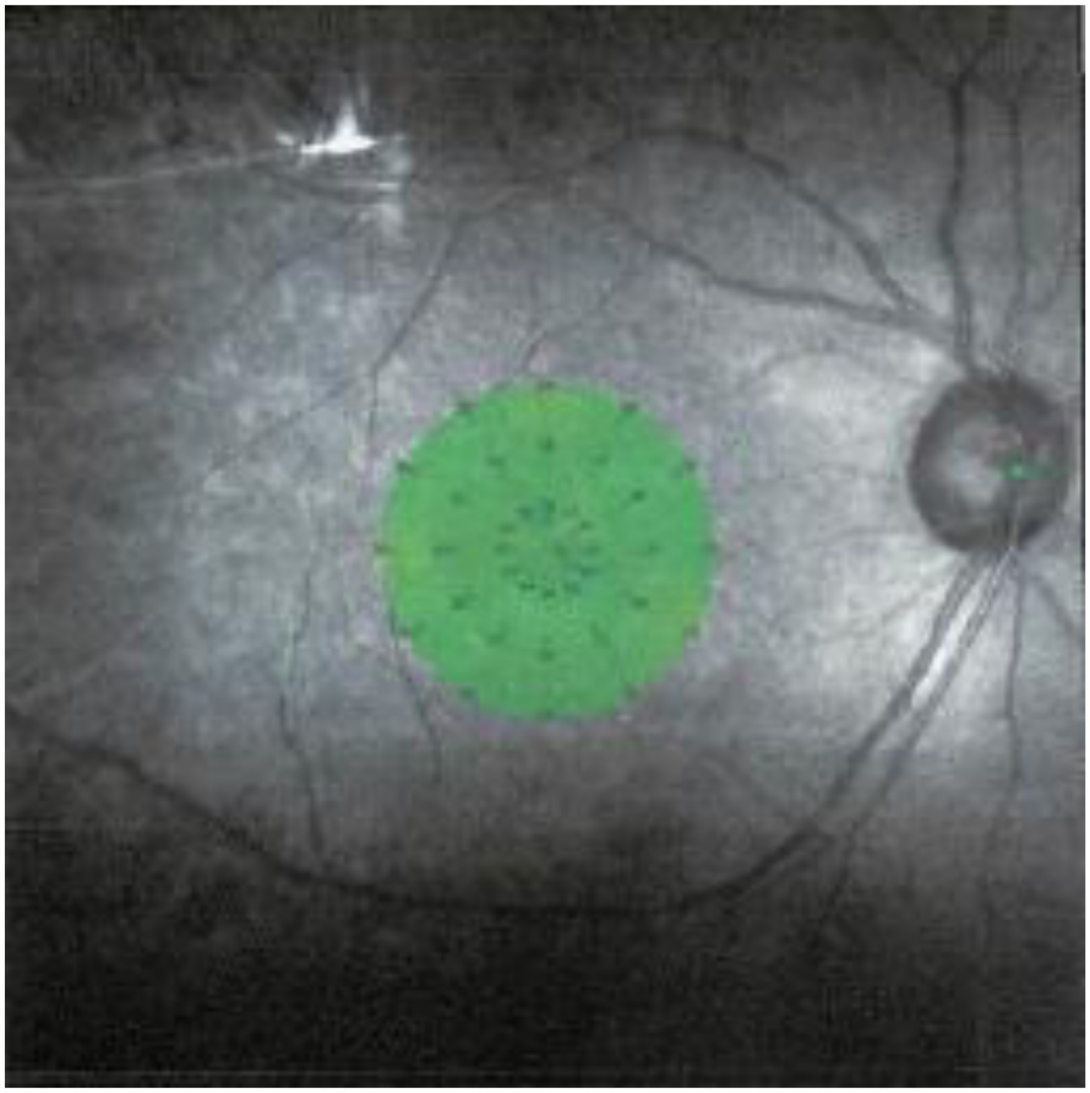
Areas of macular area assessed by Macular Integrity Assessment (MAIA) to obtain retinal sensitivity map. Thirty-seven sites within the 10° central area were assessed. The average of the 37 points was used as the retinal sensitivity for the statistical analyses.

In all examinations, Goldmann III stimuli were randomly presented according to a 4-2-0 double staircase strategy. The stimulus intensity was varied from 0 to 36 decibels (dB), the stimulus duration was 200 ms, and background illumination was set at 4 apostilb. The size of the target for fixation was varied according to the patient's visual acuity. The retinal sensitivity or average threshold (AT) was measured with the MAIA^™^ software program for the central 37 points within a 10 degree diameter (Fig 1).

### Statistical Analyses

Statistical analysis was performed using the SPSS software package (SPSS Inc., Chicago, Illinois, USA). Results are presented as the means ± standard deviations (SD). Two-way repeated measures ANOVA and a post-hoc *t* test with Bonferroni’s corrections were used to evaluate the changes in the BCVA, CMT, and AT. The Chi-square test was used to determine the significance of the changes in the integrity of the elliptical zone. The Pearson coefficient of correlation (r) was calculated to assess the association between the final BCVA after treatment and the CMT, AT, and integrity of the EZ. The strength of correlation (r value) was classified as: 0.0 to 0.2 very weak or not correlated; 0.2 to 0.4 weak or low; 0.4 to 0.7 moderate; 0.7 to 0.9 strong or high; and 0.9 to 1.0 very strong. Two-tailed *P* values <0.05 were considered to be significant.

## Results

No complications, e.g., endophthalmitis, uveitis, lens damage, cataract progression, or prolonged IOP elevation, developed that was attributable to the IVB injection.

### Improvement of retinal parameters (Fig 2)

**Fig 2:**
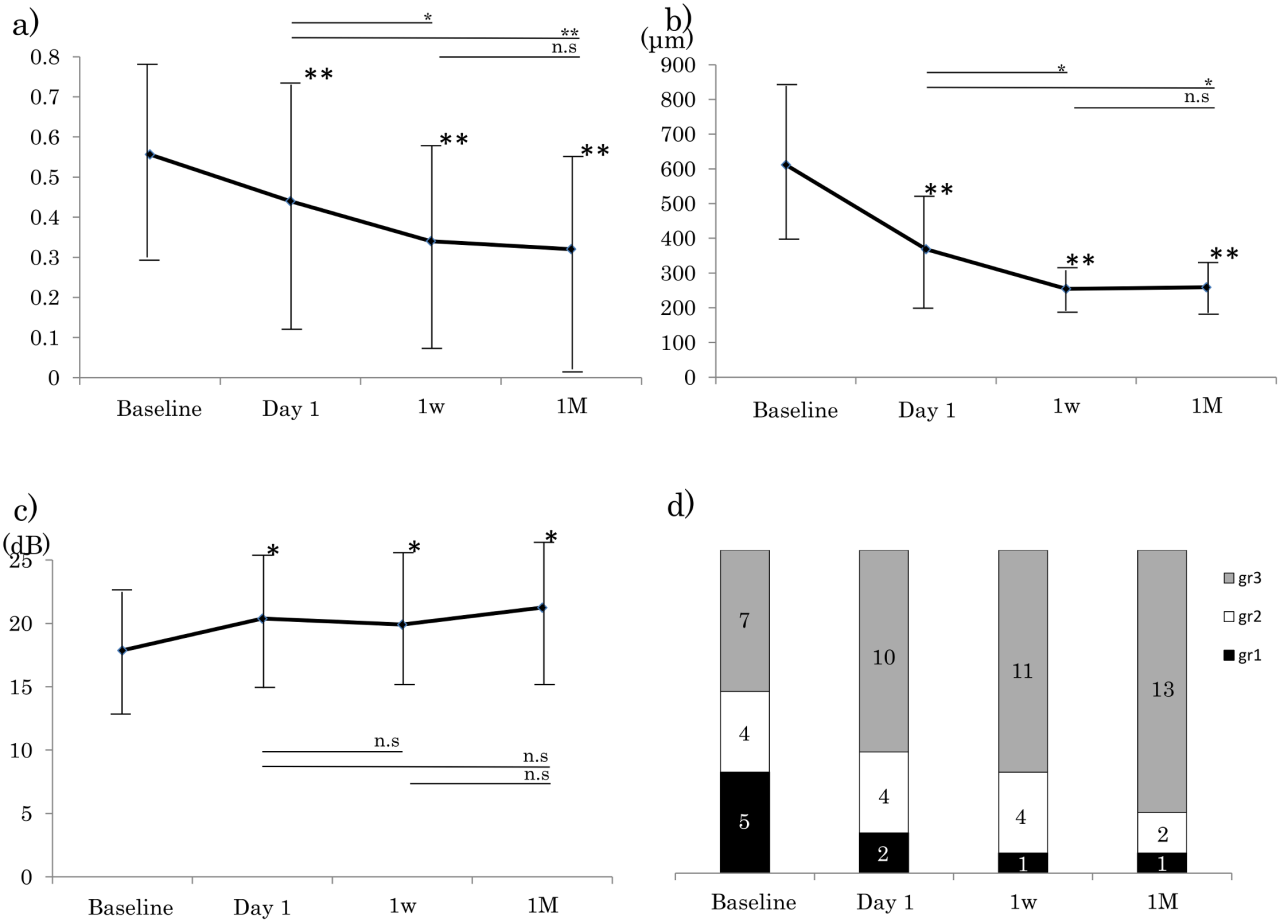
Results of assessments of morphological and functional parameters of eyes with macular edema (ME) associated with a branch retinal vein occlusion (BRVO). Assessments were made before the intravitreal bevacizumb (IVB) injection (baseline) and at 1 day, 1 week, and 1 month after the IVB. Asterisks above data indicate significant differences compared to the baseline (repeated measures ANOVA, *:*P*<0.05, **: *P*<0.01). a). Best-corrected visual acuity (logMAR units). b). Central macular thickness (CMT). c). Central retinal sensitivity which is the average of the 37 assessment sites. d). Grade of ellipsoid zone. Grey, Grade 3 = present; white, Grade 2 = abnormal or difficult to detect ellipsoid zone; and black, Grade 1 = absent.

The mean baseline BCVA was 0.56 ± 0.27 logMAR units which significantly improved to 0.44 ± 0.32 logMAR units at 1 day after the injection. The improved BCVA was maintained at 0.34 ± 0.27 logMAR units at 1week, and 0.32 ± 0.28 logMAR units at 1 month after the injection (*P* <0.05; repeated ANOVA; Fig 2a). Compared to the BCVA on day 1 after the injection, the BCVA had improved significantly at 1 week (*P* <0.05) and at 1 month (*P* <0.01). Compared to BCVA at 1 week after the injection, no significant improvement was detected at 1 month.

The baseline CMT was 611.4 ± 209.3 μm, and it decreased significantly to 368.8 ± 150.0 μm at day 1 after the injection. This decrease was maintained at 255.0 ± 42.8 μm at. 1 week, and 258.7 ± 64.0 μm at 1 month after the IVB (*P* <0.05, repeated ANOVA; Fig 2b) Compared to the CMT at day 1 after the injection, the CMT had improved significantly at 1 week and at 1 month (*P* <0.05). Compared to CMT at 1 week after the injection, no significant improvement was detected at 1 month.

The AT improved significantly from 17.9 ± 5.3 dB to 20.4 ± 6.0 dB at 1 day after the IVB. This improvement was maintained at 19.9 ± 4.7 dB at 1 week, and 21.2 ± 5.0 dB at 1 month after the injection (repeated ANOVA, *P* <0.05, Fig 2c). Compared to the AT on day 1 after the injection, the AT did not change significantly at 1 week to 1 month. Compared to the AT at 1 week after the injection, no significant change was detected at 1 month.

At the baseline, the integrity of EZ was graded as Grade 3 in 7 eyes, and the number of eyes with Grade 3 increased during the follow-up period. The integrity of the EZ had improved so that 13 eyes were graded as Grade 3(Fig 2d), but this increase in the incidence of Grade 3 was not significant (*P* = 0.28, Chi square test).

Thus, the BCVA, CMT, and AT improved significantly during the study period. The BCVA and CMT continued to improve significantly after day 1, week 1, and month 1, but the AT did not change significantly after week 1. This indicated that the AT attained its final sensitivity much earlier than the other parameters.

A representative case highlighting the association between the OCT morphology and retinal sensitivity is shown (S1 Fig).

### Correlation of BCVA and different retinal parameters (Table 1)

**Table 1:** Correlation between final best-corrected visual acuity in logMAR units and parameters at early state.

Parameter	pre			Day1			1week		
	CMT	EZ	AT	CMT	EZ	AT	CMT	EZ	AT
**Correlation coefficient**	0.18	0.52*	0.48*	0.61**	0.59*	0.76**	0.3	0.3	0.64*

*: *P* <0.05

**: *P* <0.01

CMT: central macular thickness, EZ: ellipsoid zone, AT: average retinal threshold

We calculated the correlation between the CMT, the integrity of the EZ, and the AT to the BCVA at 1 month. All parameters were moderately to strongly correlated with the BCVA at 1 month (r = 0.61 for CMT, r = 0.59 for ellipsoid zone, and r = 0.76 for AT). The AT at 1 week was moderately correlated with the BCVA at 1 month (r = 0.64).

## Discussion

Our results showed that the AT determined by MAIA^™^ and the integrity of the EZ at 1 day and the AT at 1 week after the IVB injection were significantly correlated with the BCVA. The results of earlier OCT studies showed that some morphological parameters, such as the integrity of the EZ, at the baseline were significantly associated with the BCVA after anti-VEGF injections. Our results showed that both the morphological integrity and the retinal sensitivity were significantly correlated with the BCVA at 1 month. However, the sensitivity had a higher correlation with the BCVA. Thus, both parameters can be used as assessors of the effectiveness of a specific therapy.

The assessments of the morphological alterations in the posterior pole of the eye have greatly improved with the introduction of new techniques including OCT. While various new imaging techniques are being used for morphological assessments, the functional parameters are still limited to the measurements of the BCVA and classic visual field tests. Standard fundus perimetry is time consuming and is not be used as a routine test for the growing number of patients receiving anti-VEGF treatment. Microperimetry is useful in determining the sensitivity of the central macular area and can compensate for the ambiguity of direct observations in various macular diseases [13–18]. The retinal sensitivity has been shown to be significantly correlated not only with the BCVA but also with the morphological parameters, and it can thus be used to monitor the effectiveness of treatments [20–22]. Earlier studies showed the usefulness of microperimetry, and Kriechbaum [23] reported that the correlations between the BCVA and OCT parameters for BRVO-ME during 12 months of treatment were significant. However, the experiments were performed with an older instrument. A scanning laser ophthalmoscope (SLO^®^; Rodenstock GmbH, Munich, Germany) or microperimetry-1 (MP-1^®^, NIDEK, Gamagori, Japan) was used in the earlier studies. The SLO does not have an eye tracking system and collecting the data is time consuming. Although MP-1 has an eye tracking system, it is still time consuming (about 15 min) and requires active participation by the patient. It is not that the MAIA microperimetry instrument has better sensitivity, but it has a larger dynamic range, therefore it can detect subtle changes, when dealing with higher sensitivity values. The fundus imaging method has changed from the conventional near infrared camera (MP-1) to confocal line scanning system (MAIA^™^), and this advance has enabled the recording of high resolution fundus images. For patients with unsteady fixation, such as in eyes with macular diseases, it is often difficult to evaluate the same point at different examinations. Advances in image registration by built-in confocal line scanning systems which are less affected by media opacities and better eye tracker technology make it possible to test the same area. The tracking area which is due to improvements in obtaining more secure and accurate fixation is also improved from 128 x 128 pixels (MP-1) to 1024 x 1024 pixels (MAIA^™^). These advanced eye tracker technology makes it possible to examine the same retinal area at different examinations which improves the accuracy of the assessments. In addition, the intensity range that can be tested is from 20 dB to 36 dB which greatly improves the resolution of the MAIA^™^ [19, 22].

These improvements have allowed the MAIA to be used for the early detection of age-related macular degeneration, i.e., the upper limit of sensitivity for the MP-1 attains a plateau much earlier than that with MAIA^™^, and it may fail to detect subtle changes which MAIA can detect [24]. Thus, MAIA^™^ can detect early changes and the depth of the scotoma more precisely. These technical improvements also reduced the test–retest variations. These properties of the MAIA ^™^ have allowed it to be used as a complementary objective tool for the prediction of vision outcome during or after the treatment of ME.

Many therapeutic procedures have been used to treat ME. Because prolonged ME can lead to irreversible damage of the outer retina, the BCVA can remain poor even though the ME is resolved and macular shape improved. So it is important to assess the effectiveness of the therapy as soon as possible after initiating the therapy. If a specific test indicates that the therapy is not effective, then the therapy can be switched to another. Fortunately, we have other useful choice of therapies for ME associated with BRVO such as triamcinolone acetonide [25], steroid implantation [26], and vitrectomy [27]. Jonas has reported the successful use of triamcinolone acetonide for BRVO-ME in eyes that were refractory to IVB [28]. The results of the morphological and functional parameters should provide better prognostic information so decisions can be made on whether to switch to other therapeutic procedures.

Recently, Suh and associates demonstrated that photoreceptor disruption detected by OCT was a predictor of poor visual outcome after ERM surgery, and thus prompt surgical intervention is required to prevent irreversible photoreceptor damage [29]. In our patients after the edema was resolved, the photoreceptors should recover with a recovery of the EZ. Reconstruction occurs much earlier than imaging instruments can detect, and it may affect the functional improvement as we showed by the earlier improvement of the AT on 1 day after the IVB. In addition, the integrity of EZ was often masked by the edema which prevented a quantitative evaluation. Thus, the improvements of the AT can be detected earlier than the anatomical changes.

This study has several limitations. First, we had a relatively small sample size, and the follow-up period was only 1M. However, the main disadvantage of IVB treatment for BRVO-ME is its short-term effects and high recurrences [30]. Hoh et al. reported that 65.2% of patients developed recurrences of the ME within 13.3 ± 4.4 weeks which is within the 1 month period we studied [31]. It will be difficult to follow the patients for much longer because over 1 month would be when a second injection is given if needed. If we wish to examine the long term effects, e.g., 3, 6, or 12 months, we have to take recurrences into consideration. In addition, it is necessary to determine whether our parameters can predict a recurrence or vision disturbances. Because our aim was to determine the preoperative factors that would predict the postoperative vision, we believe that an assessment at 1 month would be the most appropriate time. Different from the ME associated with central vein occlusion or diabetic macular edema, the area affected by a BRVO would be different and should be limited to a hemi-field. Thus, if we wish to evaluate the usefulness of retinal sensitivity for prediction, we have to use the AT values of the damaged area which should lead to better assessments, or we may have to evaluate the ME associated with central vein occlusion or diabetic macular edema to uniform macular dysfunction and emphasize our results. In addition, because microperimetry is a type of perimetry, there is a possibility that a learning effect may affect the results [32]. Learning effects occur even with microperimtry, and Wu et al. recommended discarding the results of the first examination to avoid the influence of a learning effect for microperimetry [33]. But as long as we examined the 10 degrees of central macular sensitivity, which we used, it is possible to avoid such test-retest variabilities [34]. To clarify this problem, we evaluated the coefficient of variation (CV, Standard deviation/average x100(%) of 7 healthy fellow eyes of our 16 patients who could perform MAIA^™^ on two different occasions. The average CV of these 7 eyes was 4.9%, and the repeatability was good although the sample number was smaller than previous reports and this was the limited results for healthy eyes. We need more careful considerations on the learning effect and test-retest variability in future studies.

Finally, it is very difficult to show differences between the AT and the BCVA. As is well known, the BCVA is the most common test of retinal function although it is subjective and reflects only the central foveal function. But, there were some of our patients whose AT value improved even though the BCVA did not improve. Other studies have reported that even standard perimetry can provide more useful information than the BCVA on the functional decrease in eyes with diabetic retinopathy or maculopathy which are also similar to BRVO-ME with perifoveal vascular damage [35, 36]. Thus, the AT obtained by microperimetry has the possibility of detecting more functional changes which the BCVA would not detect. Further investigations are needed to determine the possibility of using foveal function for prognosis.

In conclusion, the central retinal sensitivity determined by MAIA^™^ can assess the visual function after IVB treatment in eyes with ME associated with BRVO. Because these early findings are significantly correlated with the BCVA at 1 month, the MAIA^™^ -determined retinal sensitivity should be a valuable parameter to use to assess the effectiveness of therapy. The improvement of retinal sensitivity evaluation techniques has enabled clinicians to assess the effectiveness of the therapy at an early stage of treatment and make decisions on when to switch therapy.

## Supporting Information

S1 Fig Case of branch retinal vein occlusion. From the left to right, optical coherence tomographic and 37 loci central grade microperimrtric images of representative case over the follow-up (a,b: baseline and c,d: 1 weeks after treatment). Best corrected visual acuity (log MAR) before treatment was 0.3 with partially invisible ellipsoid zone (a, grade 2). White arrowheads indicates invisible ellipsoid zone. Average threshold was 21.3dB (b). One week after treatment, visual acuity was improved to 0.2 with spontaneous resolution of the macula edema and completely visible ellipsoid zone (c). Arrowheads indicates continuous ellipsoid zone. Average threshold is markedly improved to 25.0dB.
(TIF)
