# Supplementary figures and images for: Importance of Central Retinal Sensitivity for Prediction of Visual Acuity after Intravitreal Bevacizumb in Eyes with Macular Edema Associated with Branch Retinal Vein Occlusion
Source: PLoS One. 2016 Feb 17;11(2):e0149246. doi: 10.1371/journal.pone.0149246 (PMC4757563; doi:10.1371/journal.pone.0149246)

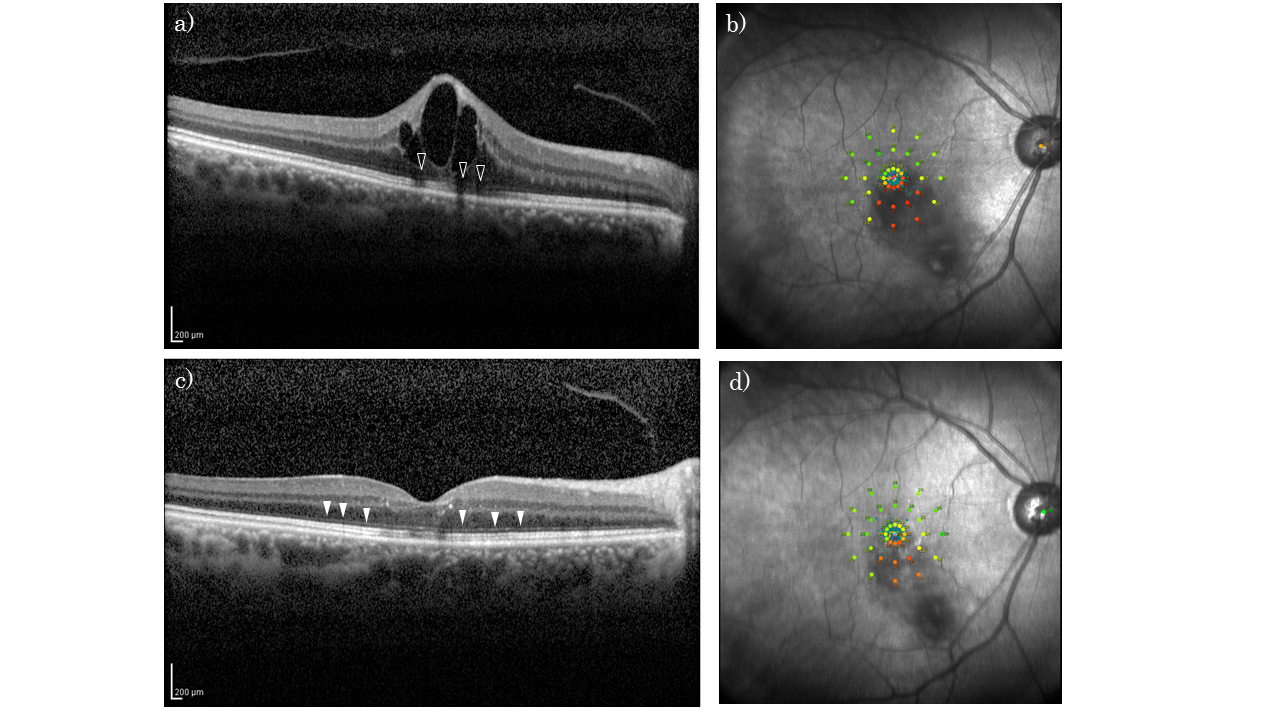

Supplement: S1 Fig — From the left to right, optical coherence tomographic and 37 loci central grade microperimrtric images of representative case over the follow-up (a,b: baseline and c,d: 1 weeks after treatment). Best corrected visual acuity (log MAR) before treatment was 0.3 with partially invisible ellipsoid zone (a, grade 2). White arrowheads indicates invisible ellipsoid zone. Average threshold was 21.3dB (b). One week after treatment, visual acuity was improved to 0.2 with spontaneous resolution of the macula edema and completely visible ellipsoid zone (c). Arrowheads indicates continuous ellipsoid zone. Average threshold is markedly improved to 25.0dB. (TIF) [file pone.0149246.s001.tif]
